# Cumulative exposure to air pollution and long term outcomes after first acute myocardial infarction: A population-based cohort study. Objectives and methodology

**DOI:** 10.1186/1471-2458-10-369

**Published:** 2010-06-24

**Authors:** Yariv Gerber, Vicki Myers, David M Broday, Silvia Koton, David M Steinberg, Yaacov Drory

**Affiliations:** 1 Dept. of Epidemiology and Preventive Medicine, School of Public Health, Sackler Faculty of Medicine, Tel Aviv University, Tel Aviv, Israel; 2 Division of Environmental, Water and Agricultural Engineering, Faculty of Civil & Environmental Engineering, Technion, Israel Institute of Technology, Haifa, Israel; 3 Dept. of Nursing, School of Health Professions, Sackler Faculty of Medicine, Tel Aviv University, Tel Aviv, Israel; 4 Dept. of Statistics and Operations Research, School of Mathematical Sciences, Faculty of Exact Sciences, Tel Aviv University, Tel Aviv, Israel; 5 Dept. of Rehabilitation, Sackler Faculty of Medicine, Tel Aviv University, Tel Aviv, Israel

## Abstract

**Background:**

Cardiovascular disease is a leading cause of morbidity and mortality worldwide and epidemiological studies have consistently shown an increased risk for cardiovascular events in relation to exposure to air pollution. The Israel Study of First Acute Myocardial Infarction was designed to longitudinally assess clinical outcomes, psychosocial adjustment and quality of life in patients hospitalized with myocardial infarction. The current study, by introducing retrospective air pollution data, will examine the association between exposure to air pollution and outcome in myocardial infarction survivors. This report will describe the methods implemented and measures employed. The study specifically aims to examine the relationship between residential exposure to air pollution and long-term risk of recurrent coronary event, heart failure, stroke, cardiac and all-cause death in a geographically defined cohort of patients with myocardial infarction.

**Methods/Design:**

All 1521 patients aged ≤65 years, admitted with first myocardial infarction between February 1992 and February 1993 to the 8 hospitals serving the population of central Israel, were followed for a median of 13 years. Data were collected on sociodemographic, clinical and environmental factors. Data from air quality monitoring stations will be incorporated retrospectively. Daily measures of air pollution will be summarised, allowing detailed maps to be developed in order to reflect chronic exposure for each participant.

**Discussion:**

This study addresses some of the gaps in understanding of the prognostic importance of air pollution exposure after myocardial infarction, by allowing a sufficient follow-up period, using a well-defined community cohort, adequately controlling for multiple and multilevel confounding factors and providing extensive data on various outcomes.

## Background

Cardiovascular disease (CVD) is a leading cause of morbidity and mortality in Israel and worldwide. The estimated incidence of myocardial infarction (MI) in Israel ranges between 20,000 and 30,000 annually [1]. Despite remarkable achievements in acute cardiac care and secondary prevention, MI patients remain a high-risk group [2]. Considering Israel's ageing population [3] and the improved survival of cardiac patients [1,2,4], an increase in CVD burden is anticipated in the following years.

Over the past two decades, epidemiological studies conducted worldwide have consistently shown an increased risk for CVD events in relation to short- and long-term exposure to air pollution [5]. Several large cohort studies have demonstrated an independent association between exposure to air pollution and cardiovascular mortality [6,7]. In a time series analysis of emergency admissions to London hospitals, Poloniecki et al. showed that MI was positively associated with black smoke, nitrogen dioxide (NO_2_), carbon monoxide (CO) and sulphur dioxide (SO_2_) and estimated that 1 in 50 cases were triggered by outdoor air pollution [8]. Furthermore, a reduction in air pollution has been associated with a significant decrease in cardiovascular deaths [9]. Exposure to traffic-related air pollution has recently been associated with daily mortality in five European cities, with a stronger effect observed in MI survivors than in the general population [10]. Among those who survived an MI, chronic exposure to particulate pollution was associated with adverse outcomes [11]. The elderly, lower socioeconomic status (SES) populations and patients with underlying cardiopulmonary disease or diabetes have been found to be particularly prone to air pollution effects [5,12].

Studies of adverse health effects related to air pollution have measured a range of compounds, including nitrates, sulphates, ozone, CO and particulate matter (PM), with fine PM, less than 2.5 μm in diameter, presenting the greatest risk to health [6,13,14]. Pope et al. [15] reported that "fine particulate air pollution is a risk factor for CVD mortality," and demonstrated that a 10-μg/m^3 ^elevation in fine PM was associated with an 8% to 18% increase in mortality risk for ischemic heart disease, dysrhythmias, and heart failure.

Although the mechanisms behind this association are not yet fully known, both animal and human studies suggest that chronic air pollution exposure may accelerate atherosclerosis [16,17]. Indeed, a population-based study demonstrated that coronary atherosclerosis was higher for participants living near a major road [18]. Air pollution from diesel exhaust has also been associated with increased thrombus formation [19].

However, methodological limitations inherent in most previous follow-up studies of MI patients challenge their applicability. The current study addresses many of these methodological problems, by defining MI with standardized clinical criteria rather than administrative data; by providing extensive information on important potential confounders (e.g., smoking, disease severity indices and SES measures); by ensuring sufficient follow-up; and by assessing multiple clinical outcomes longitudinally.

## Methods and Design

### Objective

This study will investigate the relationship between residential exposure to air pollution and long-term health outcomes in a geographically defined cohort of patients with first acute MI.

### Specific aims

**Figure 1:**
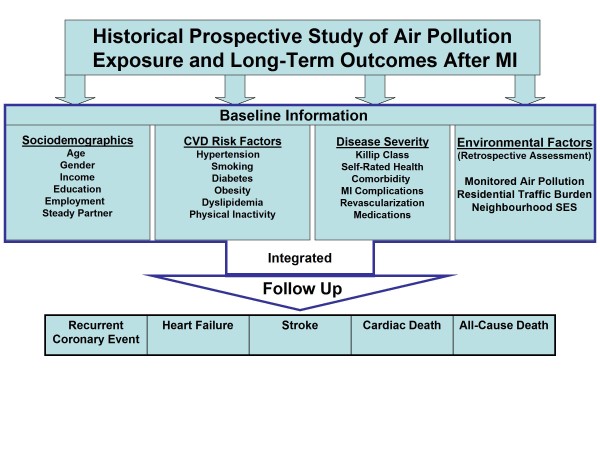
Summary of the Design Scheme

This study aims to evaluate the associations of cumulative exposure to air pollutants (NO_x_, SO_2_, and PM_x_) with the occurrence of clinical outcomes after MI, namely: recurrent coronary event; heart failure; stroke; cardiac death; and all-cause death, during a median follow-up of approximately 13 years (Figure 1). The study will assess the incremental value of air pollution exposure over (a) individual-level demographic, socioeconomic and clinical variables; and (b) neighbourhood-level SES, in predicting long-term morbidity and mortality post-MI. The effect modification of the above relationships by factors such as smoking, gender, SES, and comorbidity will be examined.

### Hypothesis

Chronic exposure to air pollutants is independently associated with long-term adverse outcomes post-MI. Heterogeneity in the magnitude of the association exists between different subsets of patients.

### Design

The current study is a historical prospective cohort study with a median of 13 years follow-up from time of incident MI. Data will be drawn from the Israel Study of First Acute Myocardial Infarction, a longitudinal prospective multicentre investigation of the role of sociodemographic, medical, environmental and psychosocial variables in long-term clinical outcomes, psychosocial adjustment and quality of life in patients hospitalized with incident acute MI over a 1-year period [20]. This study provides a wealth of data on post-MI outcomes with a relatively large sample and a high response rate, representing 98% of all non-fatal first MI cases in central Israel within the study period. Data on air pollution will be retrospectively incorporated into the already complete follow-up investigation of the parent study population.

### Study population

A geographically defined population was used. A total of 1626 patients aged ≤65 years were admitted to the 8 medical centres in central Israel with incident acute MI between February 15, 1992 and February 15, 1993. Of these, 81 (5%) died during initial hospitalization, leaving 1545 eligible candidates for the study, of which 1521 (98%) agreed to participate and completed follow-up.

### MI definition

Initial MI was defined by clinical criteria and verified by a senior cardiologist, as the presence of at least two of the following: (a) chest pain lasting at least 20 minutes;

(b) ECG changes compatible with Q wave or non Q wave MI; and (c) creatine kinase elevation ≥1.5 times the upper limit of normal or creatine kinase MB fraction >5% when simultaneous reference creatine kinase levels exceeded the upper limit of normal.

### Data collection

The entire inpatient and outpatient medical record and data obtained through structured interviews were used to ascertain demographics, SES measures, CVD risk factors, MI characteristics and severity indices, and acute management and treatment variables. Initial interviews were conducted approximately 1 week after the index hospitalization (T1). Four follow-up interviews were subsequently conducted 3-6 months (T2), 1-2 years (T3), 5 years (T4) and 10-13 years (T5) after MI.

#### Socioeconomic status characteristics

Individual SES data were self-reported at baseline and included the following measures: area of origin, family income relative to the national average (categorized as below average, average or above average), education (years of schooling), living with a steady partner, and pre-MI employment status (full-time, part-time or none; manual vs. non-manual). Income and employment status were also reported in follow-up interviews. Neighbourhood-level SES was estimated through an index developed by the Israel Central Bureau of Statistics [21], which summarizes socioeconomic measures from the 1995 National Census data, allowing the classification of small geographical units into SES categories (using a 20-point scale). Geographic Information System (GIS) tools were used to assign patients' addresses to the appropriate SES categories. Further information on neighbourhood SES [22] and individual income, employment and education [20,23] is published elsewhere.

#### Clinical variables

All medical data were reviewed, abstracted, and verified by a senior cardiologist (Y.D.). Smoking habits including intensity (number of cigarettes smoked per day), duration (years of smoking) and time since cessation were reported at T1, with the former reassessed at all follow-up interviews (T2-T5). Detailed data on smoking are published elsewhere [24]. Height and weight were recorded at T1 and again during follow-up. Obesity was defined as body mass index ≥30 kg/m^2^. Hypertension (T1 and T5) was defined as systolic blood pressure ≥140 mm Hg, diastolic blood pressure ≥90 mm Hg, or antihypertensive medication use. Diabetes mellitus (T1 and T5) was defined as (a) fasting blood glucose ≥126 mg/dl on repeated measurements; (b) 2-hour blood glucose ≥200 mg/dl following glucose loading; or (c) insulin or oral hypoglycaemic medication use and a history consistent with diabetes. Dyslipidemia (T1 and T5) was defined based on the following criteria: (a) elevated total cholesterol (>200 mg/dl); (b) elevated LDL cholesterol (>100 mg/dl); (c) low HDL cholesterol (<40 mg/dl for men, <50 mg/dl for women); or (d) elevated triglycerides (>150 mg/dl), in the context of a physician's diagnosis of dyslipidemia. Leisure time physical activity (regular, irregular or none) in the year preceding the index MI was self-reported at T1 and reassessed during follow-up (T2-T5). Comorbidity (T1) was assessed using the Charlson index [25]. MI characteristics and severity indicators included infarct type and location (T1), Killip class (T1) and left ventricular ejection fraction (T1 and T5). Revascularization procedures performed during follow-up included percutaneous coronary intervention and coronary artery bypass grafting. Medication use (e.g., aspirin, beta-blockers and statins) was recorded at baseline and during follow-up (T5). Baseline and subsequent reports of self-rated health, a 5-point scale measure (5 = excellent health), were recorded as a measure of subjective health [26].

#### Air pollution measures

Air pollution data for the study period will be retrospectively sourced from 24 air quality monitoring (AQM) stations distributed throughout central Israel. Estimates of exposure to different air pollutants will be calculated for each patient based on geo-coded residential location and proximity to a major roadway, taking into account temporal changes and changes in residential location.

Concentrations of three pollutants will be analyzed. Sulphur dioxide is emitted to the atmosphere mainly from industrial sources, in particular power plants. Nitrogen oxides are a product of combustion processes and in the study region are mostly associated with traffic-related emissions [27]. The concentrations of these two pollutants are not expected to be correlated, thereby providing independent measures of air pollution exposure [28]. Particulate matter, produced mainly by traffic and heavy industry, will also be analyzed, with emphasis on two fractions: PM_10 _(particulate matter with diameter smaller than 10 μm) and PM_2.5 _(fine particulate matter with diameter smaller than 2.5 μm).

Concentration maps for each pollutant will be created using the kriging algorithm over the greater Tel Aviv area for different averaging periods. The reliability of the maps will be assessed by a complete cross validation procedure, in which each station, in turn, will be omitted from the dataset and its records estimated by extrapolation of data from the other stations. The difference between the true and estimated results over the whole study period at each of the stations will serve as a credibility measure [29]. These average air pollution measures will be used to create an individual risk metric of exposure to pollutants for each participant.

In addition to data from the AQM stations, traffic counts will be used in circular "buffer zones" of 100 m radius around each of the reporting monitoring stations. These vehicle-specific type counts may be used within a co-kriging procedure, thus modifying the concentration maps to take account of the effect of local traffic volume on air quality.

Exposure assessment determines the extent of exposure to a hazardous agent and refers both to measurements as well as to model predictions of the fate of chemicals in the environment. Primary exposure metrics will be based on long-term average concentrations of different air pollutants. Additionally, different features of the concentration distributions (mode, median, upper percentiles, standard deviation) will be used as risk metrics, representing extreme exposures as well as the variation of exposures.

Improved risk estimation will be obtained by using additional metadata, such as the frequency with which pollutant concentrations exceeded the Israeli National Ambient Air Quality Standards (NAAQS) [30] in the nearby monitoring station during the period studied, and the distance of the patient's residence from a major roadway (following the Ministry of Transportation's road coding).

#### Outcome measures

Outcomes will include time to the following endpoints: (a) recurrent coronary event; (b) heart failure; (c) stroke; (d) cardiac death; and (e) all-cause death. Follow-up was initiated at the index MI (1992-3) and lasted through December 31, 2005 (loss to follow-up <2%). Recurrent coronary event will be composed of recurrent MI, unstable angina pectoris or cardiac death. All clinical outcomes were ascertained through various sources of data, including medical records, the Israeli Population Registry, death certificates, hospital charts, family physicians and family members and verified by a trained physician.

### Ethics

All aspects of the study have been approved by the appropriate Institutional Ethics Committees. The parent study has been approved by the Ethics Committees of all medical centres involved (Wolfson, Holon; Sheba, Tel Hashomer; Tel Aviv Sourasky, Tel Aviv; Meir, Kfar Sava; Assaf Harofeh, Zerifin; Beilinson, Petach Tikvah; Hasharon, Petach Tikvah; and Laniado, Netanya) and ratified by the Institutional Ethics Committee of Tel Aviv University both before T1 and T5. All participants gave written informed consent at both time periods. The current study, which does not involve any additional patient contact, has been approved by the Institutional Ethics Committee of Tel Aviv University.

### Sample size and power calculation

With a sample size of over 1,500 participants followed up for a median of 13 years (interquartile range 12-14 years), the average probabilities of surviving (or not experiencing the specific endpoint) to end of follow-up are as follows: 27% for recurrent coronary event; 75% for heart failure; 83% for stroke; 79% for cardiac mortality; and 72% for all-cause mortality. Table 1 summarizes the estimated statistical power for detecting a minimum hazard ratio (HR) for various outcomes in the upper quartile vs. lower quartiles of air pollution exposure, given a significance level of 5%. Comparable effect sizes (HRs ≈ 1.4) were reported in previous studies [7,11].

**Table 1:** Power Calculation for Detecting Associations Between Air Pollution Categories (Q_4 _vs. Q_1-__3_) and Primary Outcomes

	Least Extreme Detectable Hazard Ratio				
**Power**	**Recurrent Coronary Event**	**Heart Failure**	**Stroke**	**Cardiac Mortality**	**All-Cause Mortality**

80%	1.21	1.37	1.47	1.40	1.35

85%	1.23	1.40	1.50	1.43	1.37

90%	1.25	1.43	1.55	1.47	1.40

### Statistical analyses

Survival across air pollution exposure categories, estimated using the Kaplan-Meier method with right censoring at the time of last follow-up, will be compared by the log-rank test. Cox proportional hazards regression models will be constructed to evaluate the HRs for clinical outcomes associated with exposure groups (e.g. quartiles of air pollution levels) handled as time-dependent covariates. These regressions model the effects of subjects transferring from one exposure group to another during follow-up. In order to handle informative censoring in survival analyses of outcomes other than death (e.g. recurrent coronary event), proportional sub-distribution hazards regression models will be constructed accounting for death as a competing risk [31,32]. The proportionality assumption will be tested with the Schoenfeld residuals. Sequential adjustment will be made for demographic, socioeconomic and clinical variables. Since accumulating evidence suggests that an individual's health is influenced by the socioeconomic characteristics of his or her residential area, above and beyond personal SES [22,33], further adjustment for neighbourhood-level SES will be made. Hence, residual confounding of the association between air pollution and post-MI outcomes by area-level SES will be minimized. Effect modification of the above relationships by smoking, gender, SES, and diabetes will be assessed by including 2-way interaction terms (e.g. smoking-by-air pollution) in the models. Within a given air pollution-defined area, patients may be more alike with respect to unmeasured characteristics than they are between areas, therefore mixed-effects regression models will be examined, accounting for intra-area correlation [22,33,34].

As all outcome events, except for death, may have occurred more than once over the follow-up time for a given subject, ancillary analyses will be performed applying recurrent event survival analysis, with robust variance estimators computed to address the likely correlation between recurrent events in the same patient. Analyses will be performed using SAS version 9.1 (SAS Institute Inc, Cary, NC), R version 2.9 (R Development Core Team [35]), and SPSS version 18 (SPSS Inc., Chicago, IL).

### Initial Results

Participants had a mean (SD) age of 54(8) years at study entry, 19% were female, 24% were unemployed prior to MI, 47% reported a below average income and 50% had less than 12 years of education. CVD risk factors were highly prevalent at baseline, including smoking (50%), hypertension (38%) and physical inactivity (72%).

Clinical outcomes occurring during the follow-up are summarized in Table 2. Among 1521 patients, 427 deaths occurred, of which 70% were cardiac deaths.

**Table 2:** Clinical Outcomes For 1521 MI Survivors Followed Up Over 13 Years

Clinical outcome	Number of patients (%)
Recurrent MI	477 (31.4%)

Heart failure	351 (23.1%)

Ischemic Stroke	218 (14.3%)

Unstable angina pectoris (admitted)	793 (52.1%)

Cardiac death	299 (19.7%)

All-cause death	427 (28.1%)

MI, myocardial infarction

Except for death, all other clinical outcomes refer to both fatal and non-fatal events and may relate to one or more events during follow-up.

## Discussion

### Study strengths

Critics of previous studies have suggested that poorly measured confounding factors, particularly SES indicators, may account at least partly for the association between air pollution and death rates [5]. Furthermore, previous research suggests an additional contribution of residential neighbourhood to post-MI mortality risk, beyond individual SES [22]. The current study provides multiple socioeconomic measures, both individual SES and neighbourhood SES based on national census data, reflecting the multidimensional nature of this construct. Participants were included in the current study based on clinical definition of MI as ascertained by a senior cardiologist (Y.D.), as opposed to administrative or registry-based definitions criticized in previous studies.

The ethnic, cultural and socioeconomic diversity of the study population is an advantage, and the inclusion of 98% of patients aged 65 or less who survived first MI in the specified geographical area makes the results highly generalizable. The extended follow-up of 13 years provides a wealth of data, allowing a comprehensive examination of the relationship between air pollution exposure and long-term clinical outcomes in MI survivors over a period of time.

### Methodological considerations

The study is limited by the availability of air monitoring data. The air pollution index is therefore an estimate based on available data. However, maps depicting the annual average concentrations of all measured pollutants will be produced and provided that the pollutant-specific spatial patterns are consistent over time, the assessment of high-risk areas will be possible even in years with sparse data. Since some areas lack a monitoring station, data will be extrapolated from the available stations.

The heterogeneity in exposure to air pollution in the study area may be limited, since all participants resided in central Israel which, as well as covering over a third of the population, is impacted by heavy traffic and is mostly devoid of heavy industry.

Finally, the study population is a relatively "old" MI cohort, with incident MI occurring between 1992-1993. Definition of MI has since changed to include measurement of more recent cardiac biomarkers (troponins), found to be more sensitive than creatine kinase.

### Expected outcomes and contribution

As recently documented in a scientific statement from the American Heart Association concerning air pollution and CVD: "epidemiological investigations designed to address some of the limitations of prior reports are required", and include, among others: a better characterization of high-risk populations; investigation of the role of confounders; assessment of the effect of medications on the air pollution-CVD risk association; and a more thorough examination of the relationships between ambient air pollution concentrations and various adverse cardiovascular outcomes [5]. The current study presents a unique opportunity to respond to all of these recognized needs and benefits from extensive data on sociodemographics, risk factors and post-MI prognostic determinants, as well as multiple outcome measures. Limitations of previous research will be addressed by concurrently controlling for multiple and multilevel SES indicators, in addition to other important confounders such as various smoking-related parameters, disease severity indices and treatment.

This study will investigate the association between cumulative exposure to air pollution and clinical outcomes post MI and has implications for informing policy on air pollution. As recently acknowledged by experts in environmental epidemiology, "assessment of the effects of air pollution on potentially susceptible subpopulations is key to providing policy-relevant information to better protect the vulnerable" [36]. The current study addresses this issue by focusing on the hazardous effects of air pollution in a relatively large group of patients who have been suggested to be particularly susceptible to this kind of exposure.

## Competing interests

The authors declare that they have no competing interests.

## Authors' contributions

YG conceived of the study concept and design and co-drafted the manuscript. YD designed and conducted the parent study, collected data and helped revise the manuscript. DB took part in the design of the study and collected and analysed air pollution data. DS took part in the design of the study and supervised the statistical analysis. SK contributed to the design of the study and revision of the manuscript. VM drafted the manuscript. All authors read and approved the final manuscript.

## Pre-publication history

The pre-publication history for this paper can be accessed here:

http://www.biomedcentral.com/1471-2458/10/369/prepub
